# VIABAHN® Stent Graft Implantation for Iatrogenic Arteriovenous Fistula and Pseudoaneurysm of the Deep Femoral Artery

**DOI:** 10.1155/2024/3426669

**Published:** 2024-02-17

**Authors:** Daisuke Yamazaki, Takahide Fujihashi, Hirokazu Amamizu, Tomoka Kobayashi, Toru Takahashi

**Affiliations:** ^1^Department of Cardiology, Akita Cerebrospinal and Cardiovascular Center, 6-10 Senshu-Kubota-Machi, Akita 010-0874, Japan; ^2^Department of Medical Technologist, Akita Cerebrospinal and Cardiovascular Center, 6-10 Senshu-Kubota-Machi, Akita 010-0874, Japan

## Abstract

Femoral arteriovenous access is most commonly used in endovascular diagnosis and treatment. Complications arising during femoral arteriovenous access include hematoma, retroperitoneal hemorrhage, pseudoaneurysm, and arteriovenous fistula. A 66-year-old woman diagnosed with paroxysmal atrial fibrillation was treated with catheter ablation. This patient had a high femoral artery bifurcation, and we punctured the femoral vein by the conventional Merkmal method, which led to a femoral vein puncture through the deep femoral artery. The next day, echography revealed a pseudoaneurysm communicating with the deep femoral artery. We performed a thrombin injection without complication, and the pseudoaneurysm was occluded. However, echography three days after thrombin embolization showed a recurrence of the pseudoaneurysm and an arteriovenous fistula connecting to the common femoral vein. The first choice for the treatment of pseudoaneurysms and arteriovenous fistula is surgical treatment, but in addition to the lack of vascular surgery in our hospital, the patient did not want an invasive treatment and strongly preferred to be treated with a catheter. We performed endovascular treatment by VIABAHN® stent graft insertion. VIABAHN® stent graft was implanted at the deep femoral artery, and the patient was discharged without complications. VIABAHN® stent graft placement in the deep femoral artery sealed the entrance of the pseudoaneurysm and the arteriovenous fistula at once, which simultaneously treated both the pseudoaneurysm and AV fistula, and helped avoid the use of an invasive surgical procedure.

## 1. Introduction

Reportedly, 8% of femoral arteries have a high bifurcation, 2.9% have a pseudoaneurysm, and 2.2% have an arteriovenous (AV) fistula, as has been identified during femoral artery puncture during arterial catheterization procedures. Treatment of pseudoaneurysms typically includes manual compression, thrombin embolization, and surgical resection. Additionally, surgical repair is a common treatment for AV fistulas. We report a 66-year-old woman who underwent catheter ablation for paroxysmal atrial fibrillation and who developed the complication of a pseudoaneurysm and AV fistula in the deep femoral artery (DFA) due to high branching of the femoral artery. Although surgical repair for simultaneous treatment of the AV fistula and pseudoaneurysm is generally considered, we implanted a VIABAHN® stent graft in this case. The patient provided written informed consent for the report of her case details and imaging studies.

## 2. Case Presentation

A 66-year-old woman with palpitations was diagnosed with paroxysmal atrial fibrillation and was referred to our hospital for catheter ablation.

During the procedure, the right femoral vein was first punctured by the conventional Merkmal method and a dilator was inserted. Although the wire was advanced into the vein under fluoroscopy, there was arterial bleeding after insertion of the dilator, with hematoma formation. Since puncture of the right femoral vein became difficult, the left femoral vein was punctured instead and a sheath was inserted. Catheter ablation was performed with no complications except for the hematoma.

**Figure 1:**
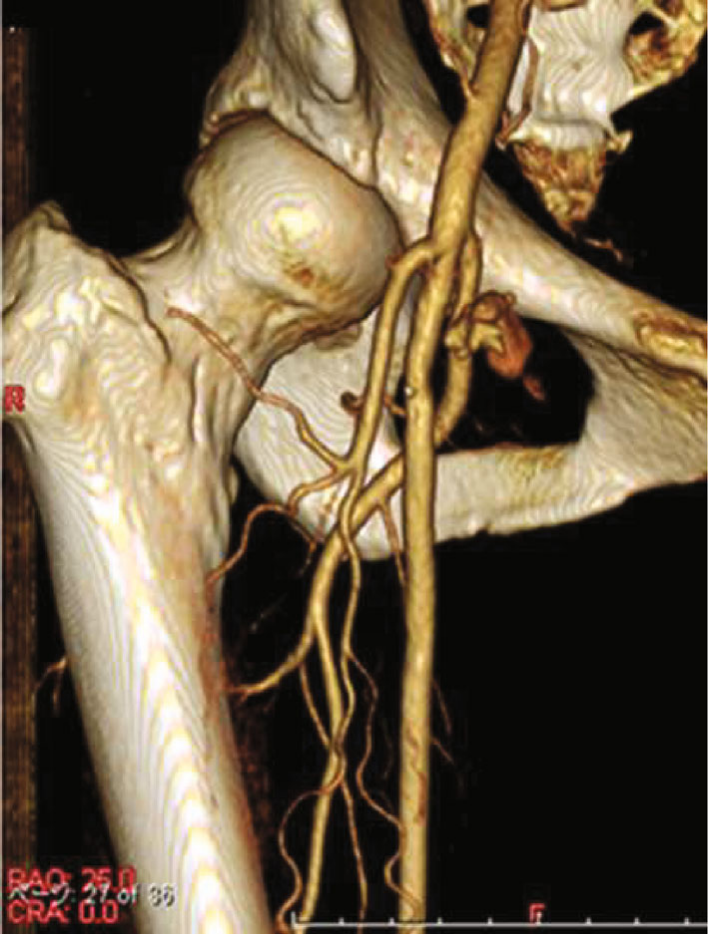
CT reveals a high femoral bifurcation and an aneurysm arising from the deep femoral artery.

However, echography performed the next day showed a pseudoaneurysm of the DFA (2.0 × 3.7 cm). The neck length of the pseudoaneurysm was 1.4 cm. Computed tomography showed a high femoral bifurcation and an aneurysm communicating with the DFA (Figure 1). At first, we tried ultrasound-guided compression of the pseudoaneurysm, but with no success.

**Figure 2:**
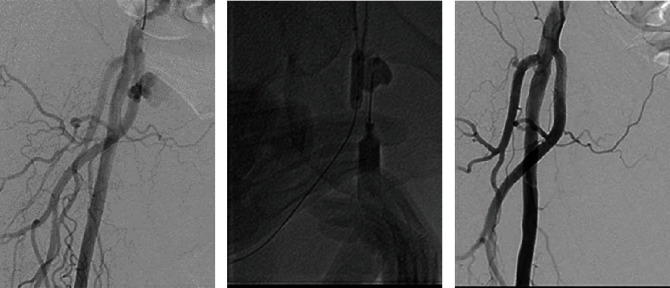
(a) Angiography shows an aneurysm arising from the deep femoral artery. (b) Balloon dilatation and thrombin injection into the aneurysm. (c) Postthrombin angiogram.

Next, we tried the injection of thrombin. A 6-Fr PARENT® sheath was inserted from the left femoral artery to the right external iliac artery, and a contrast medium was injected for visualization of the femoral artery. Angiography showed an aneurysm arising from the DFA (Figure 2). To prevent thrombin leakage into the DFA, a 6.0 mm balloon was inflated at the entrance of the pseudoaneurysm. One thousand units of a thrombin solution diluted to 50% with contrast medium was injected, so that it filled the pseudoaneurysm (Figure 2(b)). The pseudoaneurysm was punctured under echo guidance with reference to the contrast findings. The final angiogram showed no aneurysm (Figure 2(c)), and no complication occurred during the procedure.

**Figure 3:**
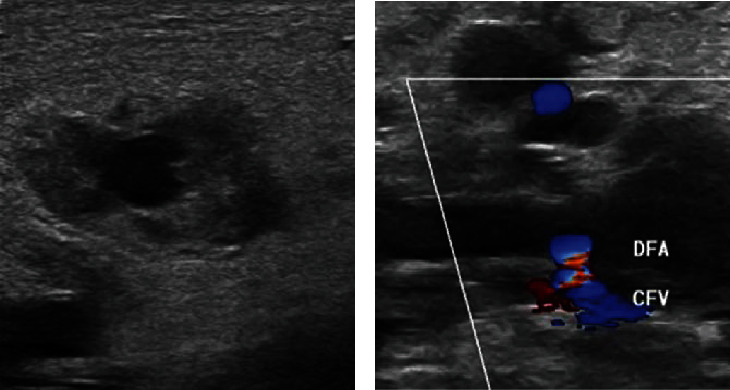
(a) Echography shows the recurrence of the pseudoaneurysm (2.1 × 4.9 cm). (b) An arteriovenous fistula is visible extending from the deep femoral artery (DFA) to the common femoral vein (CFV).

**Figure 4:**
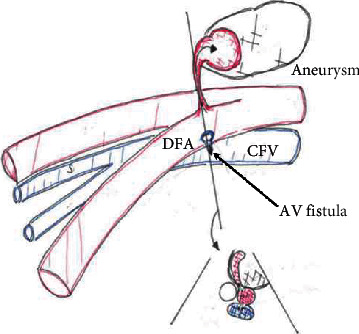
Schematic diagram by the sonographer showing the vascular relationships.

However, echography performed the next day showed a recurrence of the pseudoaneurysm (Figure 3(a)), and an AV fistula connecting to the common femoral vein was observed (Figure 3(b)). Pseudoaneurysm was slightly enlarged (2.1 × 4.9 cm).  Figure 4 is an illustration by the ultrasound technician showing the vascular relationships.

At the catheter ablation, we had inadvertently punctured the femoral vein through the deep femoral artery. Since this patient was under continued anticoagulant therapy because of her paroxysmal atrial fibrillation, the pseudoaneurysm recurred, followed by the appearance of the AV fistula.

Consequently, it was considered necessary to treat the lesion to prevent future enlargement of the pseudoaneurysm and heart failure due to the AV fistula. The patient was offered both endovascular and surgical treatment, although she preferred endovascular treatment. Therefore, we decided to implant a VIABAHN® stent graft into the DFA to simultaneously close the pseudoaneurysm and the AV fistula.

**Figure 5:**
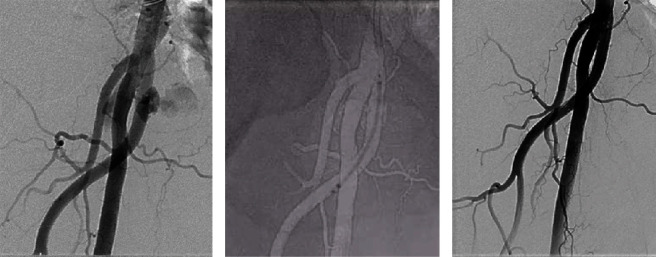
(a) Angiogram showing recurrence of the pseudoaneurysm from the deep femoral artery. (b) VIABAHN® deployment at the deep femoral artery. (c) Final angiogram.

Endovascular treatment was performed as follows. A PARENT® sheath was inserted via the left femoral artery and advanced into the external iliac artery. A 0.014-inch wire was advanced into the DFA, and the VIABAHN® (6.0 × 50 mm) was deployed in the DFA (Figures 5(a) and 5(b)).

This resulted in the complete sealing of the pseudoaneurysm and AV fistula.  Figure 5(c) shows the final angiogram. No complications occurred during the procedure. Ultrasound performed the day after endovascular treatment showed no evidence of the AV fistula or communication with the pseudoaneurysm. Continued aspirin is recommended after VIABAHN® implantation, but this patient was treated for paroxysmal atrial fibrillation in the hope that medication would be unnecessary and therefore did not wish to take aspirin either; aspirin was also discontinued after one month, and there is still no in-stent obstruction of the VIABAHN® and no recurrence of pseudoaneurysm and AV fistula. In the future, echographic follow-up is recommended to check for recurrence of pseudoaneurysms or AV fistula and obstruction in the stent graft.

## 3. Discussion

We described a case of an iatrogenic pseudoaneurysm and AV fistula in the DFA that were treated with implantation of a VIABAHN® stent graft.

The frequency of high femoral artery bifurcation has previously been reported as 8% [1]. Additionally, pseudoaneurysms were found in 2.9% of patients and AV fistula in 2.2% of patients after cardiac catheterization [2].

We usually puncture the femoral vein by the conventional Merkmal method to reduce and simplify the equipment to be prepared. In this case, the patient had a high femoral artery bifurcation. DFA bifurcated at the level of the femoral head and ran medially, which led to a puncture of the femoral vein via the DFA during puncture. Insertion of a dilator at the same site also resulted in the creation of an AV fistula.

If the puncture had been performed under ultrasound guidance, the high bifurcation might have been noticed and complications might have been prevented.

Ultrasound-guided percutaneous thrombin injection following iatrogenic pseudoaneurysm has been the typical treatment for iatrogenic pseudoaneurysms since its initial description in 1997 [3]. Anticoagulant therapy was continued postoperatively in our patient because of paroxysmal atrial fibrillation. After catheter ablation of atrial fibrillation, anticoagulant therapy should be continued to prevent mural thrombus. This resulted in the recurrence of the pseudoaneurysm and the appearance of the AV fistula.

Initially, since only a pseudoaneurysm was observed, thrombin embolization was performed as a minimally invasive treatment of the aneurysm, but since an AV fistula was also subsequently identified, thrombin embolization could not be performed. This case should have formed an AV fistula during the catheter ablation puncture and insertion of the dilator; however, there was no evidence of arterial-to-venous blood flow on echography or contrast-enhanced CT when the initial pseudoaneurysm was found. Immediately after catheter ablation, the arteriovenous shunt was closed by coagulation, and the continued anticoagulant therapy would have dissolved the coagulation and allowed the shunt to appear.

The first choice of therapy for pseudoaneurysm and AV fistula is surgical treatment; however, we do not have a vascular surgery department at our institution and we wanted to perform the least invasive treatment possible due to the patient's medical complications. Additionally, the patient refused surgical repair. We decided to insert a VIABAHN® stent graft. The patient also preferred minimally invasive treatment.

A previous case report has described the treatment of pseudoaneurysm of the popliteal artery by implantation of a VIABAHN® [4]. Another report described the treatment of a pseudoaneurysm of the DFA with VIABAHN® implantation [5]. Although placement of a VIABAHN® in the common femoral artery would have interfered with subsequent endovascular treatment, since this patient had a pseudoaneurysm and AV fistula in the DFA, we thought that placement of a VIABAHN® in the DFA would not affect future endovascular treatment.

This case is unusual in that the femoral artery was simultaneously involved with a pseudoaneurysm and AV fistula, and the lesion was in the DFA, which allowed implantation of a VIABAHN®. In this case, VIABAHN® implantation simultaneously treated both the pseudoaneurysm and AV fistula and helped avoid the use of an invasive surgical procedure.
